# Kaposi's sarcoma associated herpesvirus G-protein coupled receptor activation of cyclooxygenase-2 in vascular endothelial cells

**DOI:** 10.1186/1743-422X-4-87

**Published:** 2007-09-14

**Authors:** Bryan D Shelby, Heather L LaMarca, Harris E McFerrin, Anne B Nelson, Joseph A Lasky, Gang Sun, Leslie Myatt, Margaret K Offermann, Cindy A Morris, Deborah E Sullivan

**Affiliations:** 1 Department of Microbiology and Immunology, Tulane University Health Sciences Center, New Orleans, LA, 70112, USA; 2 Department of Medicine, Tulane University Health Sciences Center, New Orleans, LA, 70112, USA; 3 Interdisciplinary Program in Molecular and Cellular Biology, Tulane University Health Sciences Center, New Orleans, LA, 70112, USA; 4 Department of Obstetrics and Gynecology, University of Cincinnati College of Medicine, Cincinnati, OH 45267, USA; 5 Department of Hematology, Winship Cancer Institute, Emory University, Atlanta, Georgia 30322, USA

## Abstract

**Background:**

Kaposi's sarcoma associated herpesvirus (KSHV) is the etiologic agent of Kaposi's sarcoma (KS), a highly vascularized neoplasm characterized by endothelial-derived spindle-shaped tumor cells. KSHV-infected microvascular endothelial cells demonstrate increased cyclooxygenase-2 (COX-2) expression and KS lesions have high levels of prostaglandin E_2 _(PGE_2_), a short-lived eicosanoid dependent on cyclooxygenase activity that has been linked to pathogenesis of other neoplasias. To determine whether increased COX-2 expression and PGE_2 _production is mediated by the angiogenic and tumorigenic KSHV-encoded G-protein coupled receptor (vGPCR), we developed a recombinant retrovirus to express vGPCR in Human Umbilical Vascular Endothelial Cells (HUVEC).

**Results:**

In the present study, we show that vGPCR-expressing HUVEC exhibit a spindle-like morphology that is characteristic of KS endothelial cells and demonstrate selective induction of PGE_2 _and COX-2. By treating vGPCR-expressing HUVEC with selective and non-selective COX inhibitors, we show that vGPCR-induced PGE_2 _production is dependent on the expression of COX-2 but not COX-1.

**Conclusion:**

Taken together, these results demonstrate that vGPCR induces expression of COX-2 and PGE_2 _that may mediate the paracrine effects of this key viral protein in KS pathogenesis.

## Background

Kaposi's Sarcoma (KS) is a multi-cellular, highly vascularized neoplasm that is primarily composed of lymphoid, epithelial, and endothelial cells. The appearance of spindle-shaped cells, believed to be of endothelial origin, is a hallmark of KS lesions [1]. Kaposi's sarcoma associated herpesvirus (KSHV), also known as Human herpesvirus-8 (HHV-8), is the etiologic agent of both KS [2] and primary effusion lymphoma (PEL) [3] and is associated with multicentric castleman's disease (MCD) [4]. In KS, KSHV is found in spindle cells at all stages of the disease [1]. Changes in endothelial gene expression resulting from KSHV encoded gene products could provide insights into pathogenesis of KS.

KSHV has two distinct replication cycles, lytic and latent. During the lytic replication phase, infected cells express nearly all KSHV genes, including those genes required for viral DNA replication, virus packaging, and host immune response modulation to produce new virus. Latent KSHV replication is characterized by the expression of a small subset of KSHV genes that maintain infection and mediate evasion from host immune detection. Unlike lytic phase replication, viral DNA replication during latent phase is coupled to host cell replication and the latently infected cell does not produce new virus [5]. Most KSHV infected cells within KS lesions (>85%) persist in a latent state of replication [6,7].

The KSHV G-protein-coupled receptor (vGPCR) is a constitutively active lytic phase protein with significant homology to the human interleukin-8 (IL-8) receptor and has angiogenic and tumorigenic properties [8,9]. Transfection of vGPCR into endothelial and epithelial cells activates multiple transcription factors and signaling molecules including nuclear factor kappa B (NF-κB), extracellular signal regulated kinase 1/2 (ERK 1/2), p38 mitogen activated protein kinase (p38), nuclear factor of activated T cells (NFAT), c-Jun N-terminal kinase/stress-activated protein kinase (JNK/SAPK), and protein kinase C/activator protein-1 (PKC/AP-1), all of which regulate COX-2 expression [8,10-15]. The KSHV vGPCR also induces the expression of paracrine factors [8,12].

Previous studies indicate that KS lesions have increased prostaglandin E_2 _(PGE_2_) production [16] and KSHV-infected human adult dermal microvascular endothelial cells have increased cyclooxygenase-2 (COX-2) expression [17,18]. COX-2 catalyzes the conversion of arachidonic acid to Prostaglandin H_2 _(PGH_2_), a precursor for prostaglandins including PGE_2 _that are synthesized by cell-type specific prostaglandin synthases [19-21]. Human umbilical vascular endothelial cells (HUVEC) express two COX isoforms, COX-1 and COX-2, where COX-1 is ubiquitously expressed and COX-2 expression can be induced by inflammatory agents such as lipopolysaccharide (LPS) [22], and Interleukin-1-β (IL-1-β) [23], as well as mitogenic stimuli [24]. The mechanisms by which KSHV modulates expression of COX-2 and PGE_2 _in endothelial cells have yet to be associated with specific KSHV gene products.

Since vGPCR induces transcription factors known to activate COX-2 expression, we tested the hypothesis that COX-2 may be a downstream target of vGPCR intracellular signaling. Here, we demonstrate that vGPCR induces synthesis of COX-2 in HUVEC that in turn leads to PGE_2 _expression that may participate in KS pathogenesis. To our knowledge, the experiments described within this report provide the first association between a specific KSHV protein and COX-2-mediated prostaglandin production in endothelial cells.

## Results

### vGPCR induces a morphological change in primary endothelial cells

**Figure 1:**
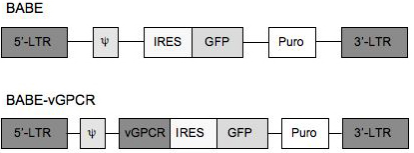
**Schematic for the retroviral expression vector**. The KSHV vGPCR is expressed as part of a bicistronic RNA upstream of an internal ribosomal entry site (IRES)-regulated GFP reporter cassette in the BABE retroviral plasmid [34]. The proviral segment of the BABE retroviral plasmid contains long terminal repeats (LTRs) that participate in proviral replication, an encapsidation sequence (ψ), and a selectable puromycin marker. This diagram is not drawn to scale.

**Figure 2:**
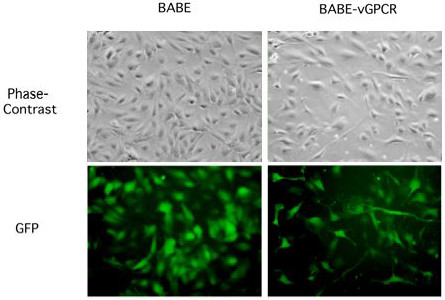
**vGPCR-expressing HUVEC mimic spindle cells**. HUVEC were infected with BABE or BABE-vGPCR retroviruses and grown for 72 hours. GFP-positive vGPCR expressing HUVEC exhibit a spindle cell-like morphology that resemble the spindle cells found in KS lesions. GFP expression ca. 60%. Images taken at 400× magnification.

To stably express the KSHV vGPCR in primary HUVEC, cells were infected with a moloney murine leukemia virus-based BABE recombinant retrovirus (BABE-vGPCR) expressing vGPCR upstream of the coding sequence for green fluorescent protein (GFP) so that the bicistronic transcript mRNA should lead to the dual expression of vGPCR and GFP (Figure 1). At 72 hours post-infection, BABE-vGPCR-infected HUVEC exhibited a spindle cell-like morphology that mimicked the endothelial-derived KS spindle cells that populate KS lesions consistent with previous reports [12,25] (Fig. 2). Conversely, infection of HUVEC with the control retrovirus, BABE, failed to induce a spindle cell-like morphology; rather, these cultures maintained a cobblestone-like appearance, which is consistent with the morphology of uninfected HUVEC. The infection efficiency for both control and vGPCR-expressing viruses was approximately 60%, as determined by quantifying GFP-positive cells using fluorescence microscopy.

### vGPCR induces expression of COX-2 but not COX-1 in primary endothelial cells

**Figure 3:**
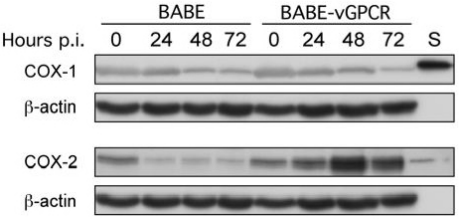
**vGPCR induces COX-2 expression**. HUVEC were infected at time 0 with either BABE or BABE-vGPCR. Whole cell lysates were prepared at the indicated time points and analyzed by western blot for the indicated protein. BABE-vGPCR-infected HUVEC demonstrate increased COX-2 expression beginning at 24 hour post-infection (p.i.), while neither BABE nor BABE-vGPCR-transduced HUVEC express COX-1. 50 ng of ovine COX-1 and COX-2 electrophoretic standards (S) served as positive controls. β-actin expression served as a loading control. Results are representative of three independent experiments.

Most cell types, including HUVEC, express two COX isoforms, COX-1 and COX-2, which perform the same function of converting arachidonic acid to PGH_2_. Sharma-Wailia et al recently demonstrated that KSHV infection of human adult dermal microvascular endothelial (HMVEC-d) cells and human foreskin fibroblasts (HFF) induced the expression of COX-2 but not COX-1 [17]. To determine whether vGPCR, alone, may regulate the expression of COX isoform expression, protein lysates of BABE or BABE-vGPCR-infected HUVEC were analyzed for COX-1 and COX-2 expression by western blot at 24-hour intervals post-infection. HUVEC infected with BABE-vGPCR displayed a time-dependent increase in COX-2 expression that began at 24 hours post-infection and remained elevated through 72 hours (Fig. 3). COX-2 expression in BABE-infected-HUVEC was identical to that in uninfected HUVEC at each time point, providing evidence that the COX-2 expression in BABE-infected-HUVEC cultures does not reflect a reduction in COX-2 expression by the retroviral control. KSHV vGPCR did not increase COX-1 expression at any time point analyzed in the experiment. Taken together, these results demonstrate that vGPCR induces expression of COX-2 but not COX-1 in primary HUVEC in a time-dependent manner.

### vGPCR induces COX-2 transcription

**Figure 4:**
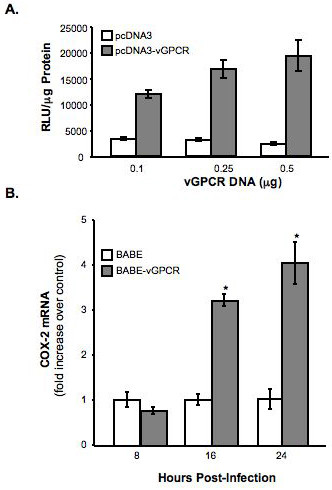
**vGPCR induces COX-2 mRNA expression**. A) A COX-2 promoter-luciferase plasmid (5 μg) was co-transfected with pcDNA3 or pcDNA3-vGPCR at the indicated concentrations. These results demonstrate a dose-dependent increase in COX-2 promoter activity in vGPCR-expressing HeLa at 48 hours (* = p < 0.001). Graph and SEM are representative of three independent experiments. B) RNA was collected from BABE and BABE-vGPCR-infected HUVEC at the indicated time points and analyzed by quantitative real-time RT-PCR for COX-2 mRNA expression. The graph demonstrates a time-dependent increase in COX-2 expression in vGPCR-expressing HUVEC and represents the mean of 3 independent infections each measured in triplicate (*= p < 0.001).

Since COX-2 expression is transcriptionally regulated in most cases, we asked whether vGPCR activated COX-2 transcription. To assess vGPCR-mediated COX-2 promoter activity, a vGPCR expression vector (pcDNA3-vGPCR) was co-transfected along with a human COX-2 promoter-luciferase construct into HeLa cells. Expression of vGPCR transactivated the COX-2 promoter in a dose-dependent manner, leading to increased luciferase activity that was 4, 5, or 6 times greater than that from cells cotransfected with the COX-2 promoter luciferase construct and pcDNA3 lacking vGPCR (Fig. 4A). The pcDNA3 vector alone displayed minimal COX-2 promoter activity at each amount analyzed in these experiments. Activation of the cellular promoter should lead to increased mRNA synthesis and this was the case. Total RNA isolated from BABE and BABE-vGPCR transduced HUVEC were analyzed by quantitative real-time RT-PCR using COX-2 specific primers. As expected, COX-2 mRNA levels in vGPCR-expressing HUVEC were 3.2 and 4-fold higher than BABE-infected HUVEC at 16 and 24 hours post infection, respectively (Fig. 4B). Collectively, these results suggest that KSHV vGPCR induces COX-2 transcription through the activation of the COX-2 promoter.

### vGPCR-expression leads to increased PGE_2 _synthesis

**Figure 5:**
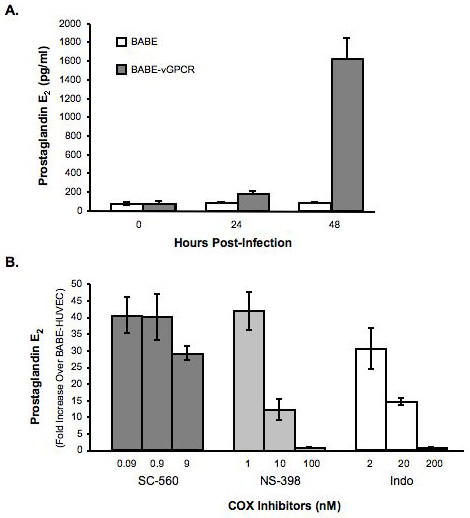
**vGPCR induced PGE_2 _secretion is dependent on COX-2**. A) HUVEC were infected with BABE or BABE-vGPCR at time 0 and analyzed at the indicated time points for the amount of PGE_2 _in conditioned medium using an EIA kit. The vGPCR-expressing HUVEC conditioned media demonstrates a time-dependent increase in PGE_2 _secretion (* = p < 0.001). Graph and SEM is representative of three independent experiments. B) At 24 hours post-infection, BABE-vGPCR infected HUVEC were treated with the non-selective COX inhibitor indomethacin (Indo), the selective COX-2 inhibitor (NS-398), or the selective COX-1 inhibitor SC-560 and PGE_2 _in the conditioned media was quantified at 48 hours post-infection by EIA. PGE_2 _secretion is reduced in a dose-dependent manner in Indo and NS-398 treated BABE-vGPCR infected HUVEC. The COX-1 selective inhibitor SC-560 had a minimal effect on PGE_2 _secreted from vGPCR expressing HUVEC. Graph indicates fold induction in PGE_2 _secretion over BABE-HUVEC and is representative of three independent experiments.

PGE_2 _synthesis is downstream of the cyclooxygenase conversion of arachidonic acid to PGH_2_. To determine whether the vGPCR-induced increase in COX-2 led to an increase in PGE_2 _synthesis, conditioned medium from BABE and BABE-vGPCR-transduced HUVEC was assayed to directly quantify secreted PGE_2 _(Fig. 5A). At 24 hours post-infection, PGE_2 _production from BABE-vGPCR-transduced HUVEC was two times greater than that from BABE-tranduced (control) HUVEC. PGE_2 _secretion from BABE-vGPCR-transduced HUVEC increased to over 20 times greater at 48 hours post-infection with the concentration of PGE_2 _exceeding 1.5 ng/ml in conditioned medium from BABE-vGPCR-transduced HUVEC. The uninfected HUVEC used in these experiments secreted low levels of PGE_2 _(50 pg/ml or less per culture) and infection with BABE did not increase PGE_2 _secretion above that detected from uninfected HUVEC (data not shown). Thus, KSHV vGPCR significantly increased PGE_2 _secretion in a time-dependent manner when expressed in primary HUVEC and, since vGPCR induces COX-2 but not COX-1 expression (Fig. 2), the increased PGE_2 _secretion was likely to result from increased COX-2 expression.

### vGPCR-induced PGE_2 _secretion requires COX-2

To confirm the role of COX-2 in vGPCR-induced PGE_2 _secretion, SC-560 (COX-1 selective inhibitor), NS-398 (COX-2 selective inhibitor), or Indomethacin (Non-selective COX inhibitor) was added to vGPCR-expressing HUVEC at 24 hours post-infection for a 24-hour treatment. The concentrations of each COX inhibitor used were based upon the published IC_50 _values: SC-560 (COX-1, 9 nM; COX-2, 6 μM), NS-398 (COX-1, 100 μM; COX-2, 0.1 μM), and Indomethacin (COX-1, 2 μM; COX-2, 20 μM) [26-28]. BABE-vGPCR-transduced HUVEC treated with either NS-398 or with indomethacin demonstrated a dose-dependent decrease in PGE_2 _secretion. In contrast, treatment with the selective COX-1 inhibitor SC-560 had no significant effect on PGE_2 _secretion of vGPCR-expressing HUVEC (Fig. 5B). These results demonstrate that the increased production of PGE_2 _in vGPCR-expressing HUVEC is dependent on vGPCR-induced COX-2 activity.

## Discussion

KS lesions are multi-cellular, highly vascularized neoplasms that express high levels of growth and inflammatory factors. There is increasing evidence that vGPCR plays a key role in KS development but the mechanisms involved are not fully understood. Aberrant induction of COX-2 and up-regulation of the prostaglandin cascade is believed to pay a significant role in carcinogenesis [29]. The data presented in this study demonstrate that KSHV vGPCR-induces COX-2 mRNA and protein expression in primary vein endothelial cells, yet has no effect on COX-1 expression. We further showed in transient expression assays, vGPCR activates COX-2 promoter-directed gene expression. The vGPCR-induced COX-2 expression results in a 20-fold increase in PGE_2 _that is significantly reduced in the presence of a COX-2 specific inhibitor. These results demonstrate a mechanism by which a lytic phase protein, vGPCR, encoded by KSHV may exert paracrine effects through COX-2 dependent prostaglandin induction.

Over-expression of COX-2 has emerged as a prominent feature of virtually every form of cancer [29]. The role of COX-2 as a critical mediator of cancer progression is supported by numerous studies showing that the induction of constitutive COX-2 expression and the resulting biosynthesis of PGE_2 _are sufficient to stimulate all of the key features of carcinogenesis including mutagenesis, mitogenesis, angiogenesis, metastasis, inhibition of apoptosis and immunosuppression [29]. KS lesions are characterized by increased levels of PGE_2 _and cyclic AMP phosphodiesterase compared to those within the surrounding tissues [16] suggesting that induction of COX-2 may be an important mediator of KS. Importantly, Sharma-Walia et al recently demonstrated that infection with KSHV induces robust COX-2, but not COX-1, expression in HMVEC and HFF and increased their secretion of PGE_2 _[17]. They further showed that although viral binding and entry induced moderate levels of COX-2, viral gene expression significantly increased COX-2 induction. To date, no specific KSHV gene products have been associated with the observed COX-2 increase. Our studies provide evidence that KSHV vGPCR induces COX-2 expression in primary vascular endothelial cells and may be responsible for the COX-2 and PGE_2 _increases observed following infection in microvascular endothelial cells and in KS clinical specimens, respectively.

Emerging evidence points to vGPCR expression as essential for KS development. It is the only KSHV gene that when expressed in the vascular endothelium of mice is able to produce vascular tumors [30] and transgenic mice that express vGPCR under either a ubiquitous (SV40) promoter or a T cell-specific (CD2) promoter also develop dermal angioproliferative lesions that closely resemble those seen in KS [31,32]. Moreover, siRNA-mediated suppression of vGPCR in mice expressing the entire KSHV genome was sufficient to block VEGF secretion and tumorigenesis, leading to significant retardation in tumor growth [33]. Whether induction of COX-2 leads to the angiogenic and tumorigenic effects of vGPCR is currently under investigation.

Increased expression of PGE_2 _due to vGPCR expression may have a role in KSHV replication as well. The study by Sharma-Walia et al suggests that COX-2 and PGE_2 _play roles in facilitating latent viral gene expression and the establishment and maintenance of latency [17]. This data together with results presented here could explain how vGPCR, a lytic gene normally expressed only in cells destined for lysis, might induce a tumor. Even though only a small percentage of KS cells express vGPCR within KS lesions, vGPCR induced COX-2 expression and consequent PGE_2 _secretion could initiate tumorigenesis and promote viral latency through paracrine mechanisms. There are no published reports describing a clinical link between COX-2 and KS, however, we feel that this hypothesis warrants further investigation.

COX inhibitors such as aspirin inhibit COX activity by blocking the conversion of arachidonic acid to PGH_2 _by competing with free arachidonic acid for the cyclooxygenase active sites. The COX-1 and COX-2 pharmacological inhibitors NS-398, SC-560, and Indomethacin used in this study abrogate cyclooxygenase activity by a similar mechanism. The use of COX-2 inhibitors would provide a viable therapeutic strategy to abrogate PGE_2 _secretion since prostaglandin E synthase (PGES) cannot synthesize PGE_2 _without PGH_2 _and there are no direct inhibitors of PGES currently available. Given the correlation of COX-2 in other cancer models, and evidence that regular intake of a COX-2 inhibitor reduces cancer risk [29], future investigations into the mechanisms of KSHV induced COX-2 expression and prostaglandin activity may lead to new treatments for KS patients.

## Methods

### Reagents

NS-398 was purchased from Cayman Chemical, Ann Arbor, MI and sodium butyrate (NaB) was purchased from Sigma, St. Louis, MO.

### Cell culture

Pooled HUVEC (Cambrex BioScience, Walkersville, MD) were cultured on 0.2% gelatin coated plates in medium 199 (M-199) (Invitrogen, Carlsbad, CA) supplemented with 20% fetal bovine serum (FBS), 2 mM L-glutamine, 2 mM penicillin-streptomycin, and 1% endothelial cell growth supplement (ECGS) (BD Biosciences, Bedford, MA). Phoenix GP retroviral packaging cells (ATCC, Manassas, VA) were grown in Dulbecco's modified essential medium (DMEM, Invitrogen) supplemented with 10% FBS, 2 mM L-glutamine, and 2 mM penicillin-streptomycin. HeLa cells (ATCC) were grown in minimal essential media supplemented with 10% FBS, 2 mM L-glutamine, 2 mM penicillin-streptomycin, non-essential amino acids, and sodium pyruvate. All cells were grown at 37°C with 5% CO_2_.

### Plasmids

To construct vGPCR-expressing plasmids, KSHV vGPCR cDNA was cut from MIGR-ORF74 (generous gift from Marvin Reitz, Institute of Human Virology, Baltimore, MD) by *BglII *and *EcoRI *digestion [12]. The digested vGPCR fragment was separated by gel electrophoresis, purified using QIAquick PCR Purification Kit (Qiagen, Valencia, CA) and ligated into either *BamHI *and *EcoRI *digested pBABE-green fluorescent protein (GFP) retroviral plasmid (generous gift from Andrew Rice, Baylor University, Houston, TX) [34] or *BamHI *and *EcoRI *digested pcDNA3 (Invitrogen) using T4 DNA Ligase (Invitrogen). pBABE-GFP and pBABE-GFP-vGPCR were propagated in *Escherichia coli *strain STBL2 (Invitrogen), whereas pcDNA3-vGPCR, pcDNA3, and plasmids containing the full-length COX-2 promoter upstream of luciferase (generous gift of Nicholas Bazan, Louisiana State University Health Sciences Center, New Orleans, LA) or vesicular stomatitis virus G envelope protein (pVSV-G, generous gift of Gary Nolan, Stanford University, Palo Alto, CA) were propagated in *Escherichia coli *strain DH5α (Invitrogen). All plasmids were harvested using QIAfilter Plasmid Maxi Kit (Qiagen) according to the manufacturer's instructions and quantified by spectrophotometry.

### Retrovirus Production and Infection

BABE(VSV-G) pseudotypes were produced by transfecting Phoenix GP cells with an equal amount of either pBABE-GFP or pBABE-GFP-vGPCR and pVSV-G using calcium phosphate precipitation as previously described [35]. The medium was removed from each culture at 15 hours post-transfection, cells were washed twice with phosphate-buffered saline (PBS), and DMEM supplemented with 5 mM NaB was added. Fourteen hours later, the cells were washed twice with PBS and the medium was changed to M-199 supplemented with 10% FBS. At 48 hours post-transfection, the virus-containing medium was collected, centrifuged at 200 *x g *for 10 minutes to remove debris, passed through a 0.45-μm filter, aliquoted and stored at -80°C. Virus titers were quantified by GFP expression in retrovirus-transduced NIH3T3 cells (ATCC) using fluorescent microscopy. HUVEC between passages 4 and 6 were plated 24 hour prior to infection at a confluence of 40%. At the time of infection, the medium was replaced with BABE or BABE-vGPCR retrovirus-containing medium diluted in M-199 (20% FBS) with ECGS to an MOI of 0.5. The virus-containing medium was removed 24 hour post-infection and fresh M-199 (20% FBS) with ECGS was added every 24 hours for the remainder of each experiment.

### Western Blot Analysis

Whole cell extracts were harvested in RIPA Buffer (50 mM Tris-HCl pH 7.5, 1% Nonidet P-40, 150 mM NaCl, 0.5% sodium deoxycholate, 0.1% SDS, 1 mM phenylmethylsulfonylflouride, 1 mM sodium orthovanadate, 1 mM sodium flouride, and 10 μg/ml aprotinin), rotated for 30 min at 4°C, and centrifuged at 14,000 rpm for 15 min. Clarified protein was quantified by Bradford assay (Sigma). 45 μg of protein from each sample was separated by SDS-PAGE, transferred to a PVDF membrane, and incubated overnight with COX-1 or COX-2 monoclonal antibody (Cayman Chemicals, Ann Arbor, MI) diluted 1:1000 in 5% non-fat milk/0.1% Tween-TBS. After incubation with an anti-mouse secondary antibody conjugated with horseradish peroxidase (1:5000 dilution, Sigma), the immunocomplexes were visualized by enhanced chemiluminescence (Amersham Biosciences, Buckinghamshire, England). β-actin expression was measured as a loading control using an anti-β-actin rabbit polyclonal antibody (1:1000) (Sigma) and detected by a 1:5000 dilution of horseradish peroxidase conjugated donkey anti-rabbit antibody (Amersham Biosciences).

### PGE_2 _Quantification

Conditioned medium from BABE and BABE-vGPCR transduced HUVEC was centrifuged at 14,000 rpm for 15 min at 4°C and supernatants assayed immediately using a monoclonal PGE_2 _EIA kit (Cayman, Ann Arbor, MI) according to the manufacturer's protocol.

### Luciferase Assay

HeLa cells were co-transfected in duplicate with 5 μg of full-length COX-2 promoter luciferase plasmid and increasing amounts of either pcDNA3 or pcDNA3-vGPCR using calcium phosphate precipitation as previously described [35]. Medium was removed 18 hours post-transfection and replaced with fresh culture medium. At 48 hours post-transfection, medium from each well was replaced with Luciferase Lysis Buffer (Promega, Madison, WI) and incubated at -80°C for 10 min. Each sample was thawed to room temperature, scraped, and centrifuged at 14,000 rpm for 15 min at 4°C. Clarified protein for each sample was quantified by Bradford assay (Sigma) according to the manufacturer's protocol. Luciferase Reporter Buffer (Promega) was added to each sample and relative luciferase activity was measured in triplicate using a luminometer (Lumat LB9507, Berthold).

### Real-Time Reverse Transcriptase PCR

Total cellular RNA was isolated using RNeasy Total RNA Kit (Qiagen) according to the manufacturer's instructions. DNA was eliminated from all samples using Turbo-DNase I (Ambion, Austin, TX). The RNA from each sample was quantified by spectrophotometry. RNA (250 ng) from each sample was converted to cDNA using iScript Reverse Transcriptase (RT) (Bio-Rad, Hercules, CA) following the manufacturer's protocol. Two microliters of cDNA was amplified in 20 μl reactions containing primers at 250 nM in iQ SYBR Green Supermix (Bio-Rad). PCR was performed for 40 cycles consisting of 95°C for 15 sec and 60°C for 45 sec using an iCycler iQ Real Time Detection System (Bio-Rad) using primers specific for COX-2 mRNA [36] and human riboprotein 36B4 [37]. Dilution curves showed that PCR efficiency was 96–100% for all primer sets used. All samples were run in triplicate on the same plate for each primer set. Negative controls, such as cDNA reactions without reverse transcriptase or RNA, and PCR mixtures lacking cDNA were included in each PCR to detect possible contaminants. Following amplification, specificity of the reaction was confirmed by melt curve analysis. Relative quantitation was determined using the comparative C_T _method with data normalized to 36B4 mRNA and calibrated to the average ΔC_T _of untreated controls.

### Statistical analysis

Data are presented as the means +/- standard error of the means (SEM). Data from vGPCR-expressing groups were compared to control groups and significant differences were determined by one-way analysis of variance (ANOVA) followed by Tukey's post hoc t-test (GraphPad Prism Home, San Diego, CA).

## Competing interests

The author(s) declare that they have no competing interests.

## Authors' contributions

BDS participated in experimental design, implementation, interpretation of results and drafting the maunscript. HLL helped with real-time PCR analyses. HEM helped with retroviral production. ABN performed western blot analyses. JAL participated in experimental design. GS and LM provided expertise in real-time PCR analyses. MKO participated in experimental design and data interpretation. CAM participated in experimental design, data interpretation and manuscript preparation. DES participated in experimental design, data interpretation and manuscript preparation. All authors read and approved the final manuscript.
